# Effects of Bacterial Nanocellulose Loaded with Curcumin and Its Degradation Products on Human Dermal Fibroblasts

**DOI:** 10.3390/ma13214759

**Published:** 2020-10-25

**Authors:** Marketa Zikmundova, Maria Vereshaka, Katerina Kolarova, Julia Pajorova, Vaclav Svorcik, Lucie Bacakova

**Affiliations:** 1 Department of Biomaterials and Tissue Engineering, Institute of Physiology of the Czech Academy of Sciences, 142 20 Prague, Czech Republic; Julia.Pajorova@fgu.cas.cz (J.P.); lucie.bacakova@fgu.cas.cz (L.B.); 2 Department of Solid State Engineering, Faculty of Chemical Technology, University of Chemistry and Technology, 166 28 Prague, Czech Republic; mashunya59@yandex.ua (M.V.); kolarovak@fzu.cz (K.K.); vaclav.svorcik@vscht.cz (V.S.)

**Keywords:** bacterial nanocellulose, curcumin, curcumin thermal degradation, dermal fibroblasts

## Abstract

Bacterial nanocellulose has found applications in tissue engineering, in skin tissue repair, and in wound healing. Its large surface area enables the adsorption of various substances. Bacterial nanocellulose with adsorbed substances can serve as a substrate for drug-delivery of specific bioactive healing agents into wounds. In this study, we loaded a bacterial nanocellulose hydrogel with curcumin, i.e., an important anti-bacterial and healing agent, and its degradation products. These products were prepared by thermal decomposition of curcumin (DC) at a temperature of 180 °C (DC 180) or of 300 °C (DC 300). The main thermal decomposition products were tumerone, vanillin, and feruloylmethane. Curcumin and its degradation products were loaded into the bacterial nanocellulose by an autoclaving process. The increased temperature during autoclaving enhanced the solubility and the penetration of the agents into the nanocellulose. The aim of this study was to investigate the cytotoxicity and the antimicrobial activity of pure curcumin, its degradation products, and finally of bacterial nanocellulose loaded with these agents. In vitro tests performed on human dermal fibroblasts revealed that the degradation products of curcumin, i.e., DC 180 and DC 300, were more cytotoxic than pure curcumin. However, if DC 300 was loaded into nanocellulose, the cytotoxic effect was not as strong as in the case of DC 300 powder added into the culture medium. DC 300 was found to be the least soluble product in water, which probably resulted in the poor loading of this agent into the nanocellulose. Nanocellulose loaded with pure curcumin or DC 180 exhibited more antibacterial activity than pristine nanocellulose.

## 1. Introduction

Cellulose is the most abundant natural polymer. Various natural fibers, e.g., cotton and higher plants, have cellulose as their main component [1]. Cellulose has found application in various industrial and biotechnological fields due to its availability, low cost, non-toxicity, environmentally-friendly behavior, biodegradability, and also thermal and chemical stability [2]. As a natural polymer, cellulose, including nanocellulose, is usually obtained from natural sources such as plants, bacteria, algae, and animals (for a review, see [3]). Bacterial nanocellulose is deposited extracellularly by various aerobic Gram-negative bacteria. In industrial applications, the *Acetobacter* or *Gluconacetobacter* genus is used due to their high yield production [4]. Bacterial cellulose is chemically identical to plant cellulose, consisting of a linear chain of several hundred to thousands of glucose units. However, bacterial cellulose is free of plant bioproducts such as lignin, pectin, hemicelluloses, and other metabolic substances, and this brings advantages in biomedical applications [5,6].

Bacterial nanocellulose has found applications in tissue engineering, in skin tissue repair, and in wound healing. Nanocellulose has important properties for these applications, mainly its appropriate mechanical strength (tensile strength, Young’s modulus, elasticity) and high water-absorbing capacity, which ensures the retention of moisture in the damaged skin, and, at the same time, adsorption of the exudate from the wounds [3]. Bacterial cellulose hydrogels have shown great ability to stimulate wound healing. For example, Kaminagakura et al. (2019) successfully healed full-thickness skin defects in guinea pigs by a bacterial nanocellulose membrane under the trade name Nanoskin^®^ [7]. Bacterial cellulose consists of fine nanofibrils. This nanoscale morphology mimics the nanoscale architecture of the natural extracellular matrix, and nanocellulose can therefore be regarded as a suitable substrate for the adhesion and growth of skin cells. The drawback of cellulose is its non-degradability in the human organism, which limits the use of cellulose as a scaffold for cells in skin replacement [3]. Nevertheless, cellulose can be used as a temporary carrier for delivering skin cells into wounds [8,9]. In this process, the cells seeded on the carrier adhere to the wound bed, migrate into the wound and colonize it, and finally the carrier can be removed. In our previous study, we used a plant-derived carboxymethylcellulose microfibrous membrane coated with a fibrin nanofibrous mesh and pre-seeded with skin fibroblasts in order to create a cell carrier with the potential to deliver skin cells into cutaneous wounds [10]. In addition, the large surface area of bacterial nanocellulose enables the adsorption of various ions, molecules, and substances, and it can therefore serve as a substrate for drug-delivery of specific bioactive healing agents into wounds. 

An important property of wound dressings should be the ability to protect wounds from microbial infections, which are caused mainly by bacteria. Bacterial nanocellulose without any additives is not usually able to inhibit bacterial growth. Therefore, many studies have dealt with incorporating bacterial cellulose with various antibacterial agents, e.g., silver nanoparticles, antibiotics, antiseptics, and various nature-derived antibacterial molecules [3]. In our study, we loaded bacterial nanocellulose with curcumin, a polyphenolic yellow pigment isolated from *Curcuma longa*. Several studies have reported its broad spectrum of antimicrobial activity, mainly against bacteria, viruses, and fungi (for a review see [11]). Moreover, curcumin is considered as a potent anti-inflammatory, anti-oxidative, and anti-carcinogenic agent [12,13]. Curcumin has also been reported to contribute to wound healing by supporting the proliferation and migration of fibroblasts into the wound, collagen deposition by these cells, wound contraction, re-epithelialization, and tissue remodeling [14].

The applications of curcumin are limited by its extremely low water solubility, which leads to its poor bioavailability. In general, curcumin is almost insoluble in polar solvents. In many studies, organic solvents such as ethanol [15] or dimethyl sulfoxide [5] have been used for curcumin dissolution [13]. Curcumin is unstable in physiological and alkaline pH, and degrades mainly to ferulic acid, feruloylmethane, and vanillin [16]. Curcumin undergoes rapid non-enzymatic degradation in cell culture media, and it is metabolized quickly in vivo [17]. Furthermore, curcumin is susceptible to degradation by ultraviolet (UV) light [12] and also to thermal degradation. Esatbeyoglu et al. (2015) studied the thermal stability of curcumin and proved that roasting curcumin led to its degradation, with significant impacts on various types of cells [13]. In addition, heat treatment of curcumin can markedly increase its solubility in aqueous solvents, and thus its bioavailability [18]. Other methods for increasing the solubility and the bioavailability of curcumin include encapsulation of curcumin into water-soluble molecules, e.g., cyclodextrins [19], or formulating curcumin into various drug-delivery systems [5,15]. For example, Guo et al. (2017) loaded curcumin onto composite scaffolds consisting of gelatin microspheres and porous collagen/cellulose nanocrystals to show strong antibacterial activity and dermis regeneration ability in rats with full-thickness skin burns [15]. Curcumin is mainly applied with plant-derived nanocellulose in the form of a wound dressing. The combination of curcumin with bacterial cellulose is relatively rare. In a recent study, Khamrai et al. (2019) entrapped curcumin into a polymeric hydrogel composite containing gelatin and ionically modified self-assembled bacterial cellulose to create a smart drug delivery system. These authors observed the wound healing activity of this system together with its antimicrobial effect [5]. Other recently developed systems for increasing the bioavailability of curcumin include Pickering emulsion-stabilized nanocellulose-based nanoparticles for curcumin encapsulations [20], nanocellulose-reinforced chitosan hydrogel incorporated with Tween 20 [21], polyvinyl alcohol/polyethylene oxide/carboxymethyl cellulose matrix blended with nanosilver nanohydrogels, *Aloe vera* and curcumin deposited on a hydrolyzed polyethylene terephthalate fabric [22], or electrospun nanofibers containing poly(lactide-*co*-glycolide), cellulose nanocrystals, curcumin, and polyethyleneimine-carboxymethyl chitosan/pDNA-angiogenin nanoparticles [3,23]. These systems are advanced and sophisticated but are rather complicated. It is therefore always reasonable to search for more easily available and more efficient systems for curcumin delivery.

Therefore, the first aim of this study was to utilize the increased water solubility of thermally-treated curcumin for its incorporation into bacterial nanocellulose in the form of a hydrogel. We prepared a bacterial nanocellulose hydrogel loaded with curcumin or with curcumin degradation products obtained by thermal decomposition of the curcumin molecule at temperatures of 180 °C and 300 °C. The second important aim of our study was to investigate the cytotoxicity and the antimicrobial activity of pure curcumin, curcumin degradation products, and also of bacterial nanocellulose loaded with these agents. In order to further enhance the water solubility and the penetration of curcumin and its degradation products into the bacterial nanocellulose hydrogel, the agents were loaded by an autoclaving process that enabled temperature control. At the same time, autoclaving ensured sterilization of the nanocellulose samples.

## 2. Materials and Methods 

### 2.1. Preparation of Bacterial Nanocellulose and Curcumin Degradation Products

Bacterial nanocellulose hydrogel was obtained by employing a bacterial strain of *Komagatabacter sucrofermentans* that synthesizes nanocellulose during its cultivation. This DSM 15973 strain was purchased from Leibniz-Institut DSMZ, Braunschweig, Germany. Cultivation was carried out in static mode in Hestrin–Shramm culture medium. The medium consisted of D-glucose (20 g/L), disodium hydrogen phosphate dodecahydrate (6.8 g/L), special peptone (5 g/L), yeast extract (5 g/L), and citric acid monohydrate (1.3 g/L). Cultivation lasted for at least 7 days at 28 °C without shaking. The nanocellulose was purified from bacteria by rinsing in water, then in boiling 0.1 M NaOH, in boiling distilled water, and finally in distilled water.

Curcumin powder with molecular weight M = 368 g∙mol^−1^ (Sigma-Aldrich, Bangalore, India) was degraded by heating it up in a BINDER chamber dryer (Binder GmbH, Tuttlingen, Germany) at a degradation temperature of 180 °C or 300 °C. Thermal degradation was performed for 2.5 h. After being heated up, the degraded curcumin was cooled at laboratory temperature. 

### 2.2. Morphology of Bacterial Nanocellulose

The surface structure and the morphology of the nanocellulose was studied using scanning electron microscopy (SEM). The scanning was performed on a VEGA 3 MLU (TESCAN, Brno, Czech Republic). The measurements were conducted by a secondary electron (SE) detector. The micrographs were taken at an acceleration voltage of 5 kV.

### 2.3. Investigation of Thermal Decomposition of Curcumin

The thermal decomposition, manifested by a change in the mass of the curcumin, was studied by thermogravimetric analysis (TGA). Fourier-transform infrared spectroscopy (FT-IR) was used for identifying the characteristic chemical functional groups. Gas chromatography with a mass spectrometer (GS–MS) was used for a detailed study of the components obtained by thermal decomposition of the curcumin powder. 

TGA was performed on a TG750 device (Stanton Redcroft Ltd., London, United Kingdom). The rate of heating was 10 °C/min in an oxygen atmosphere. The initial mass of the curcumin powder was 2.428 mg. The change in the mass of the curcumin was expressed as TGA and dTG (first derivative of the TGA curve) curves.

FT-IR analysis was conducted on a NICOLET 6700 spectrometer with an attenuated total reflection (ATR) diamond crystal GradiATR. FTIR spectra were measured in the range of 400–4000 cm^−1^ with a spectral resolution of 4 cm^−1^ and was evaluated with Omnic Software (all from ThermoFisher SCIENTIFIC, Waltham, MA, USA). 

GS-MS was performed on an Agilent 7890 GC with an Agilent 7200 Q-TOF and an Agilent Thermal Separation Probe (Agilent, Santa Clara, CA, USA) mounted on the multi-mode inlet. The samples were desorbed at 80 °C in a helium atmosphere, and the products were analyzed on-line by the GC–MS method in order to determine traces of semivolatile organic compound present in the powder samples.

### 2.4. Loading Curcumin and Its Degradation Products into Bacterial Nanocellulose

Curcumin, curcumin degraded at 180 °C (DC 180), and curcumin degraded at 300 °C (DC 300) into bacterial nanocellulose were loaded from solutions of these agents in deionized water. For each agent, solutions with different concentrations were prepared, namely 0.05, 0.1, and 0.5 mg/mL. The agents were loaded into the nanocellulose by autoclaving (121 °C, 20 min; Tuttnauer Steam Sterilizer 2540 ELC, Hauppague, NY, USA) the nanocellulose in the agent solutions. Autoclaving increased the water solubility and the penetration of the agents into the nanocellulose. In addition, autoclaving ensured that the nanocellulose samples were sterilized. After autoclaving, the nanocellulose samples were rinsed in deionized water (equipment from GORO, spol. s r.o., Brandýs nad Labem, Czech Republic) at 37 °C. 

### 2.5. Cytotoxicity of the Materials

The potential cytotoxicity of curcumin, the DC 180 and DC 300 powders, and nanocellulose loaded with these agents was investigated using human neonatal dermal fibroblasts purchased from Lonza (Basel, Switzerland, Cat. No. CC-2509).

For an investigation of the potential cytotoxicity of curcumin, DC 180 and DC 300, we prepared 5 mg/mL solutions of these agents in deionized water. The solutions were autoclaved (121 °C, 20 min) in order to ensure the same conditions as those under which the agents were loaded into the nanocellulose hydrogel. The solutions were then diluted with the cell cultivation medium, namely Dulbecco’s modified Eagle’s medium (DMEM; Sigma-Aldrich Co., St. Louis, MO, USA), supplemented with 40 µg/mL of gentamicin (LEK, Ljubljana, Slovenia) in order to obtain various concentrations of the agents in this medium. These concentrations were 0.01, 0.05, 0.1, and 0.5 mg/mL. Finally, fetal bovine serum (FBS; Sebak GmbH, Aidenbach, Germany) in 10% concentration was added to the solutions of the agents in the cultivation medium. The solutions of curcumin, DC 180 and DC 300 in the cultivation medium (0.5 mL) were added to cells cultivated on the bottom of wells in 48-well cell culture plates (TPP, Trasadingen, Switzerland, growth area 0.95 cm^2^). The cells were seeded 24 h before adding the medium with the tested agents at a density of 10^3^ cells/well into 0.5 mL of DMEM supplemented with 10% of FBS and 40 µg/mL of gentamicin. Cells cultivated in DMEM with 10% of FBS and 10% of deionized water were used as a control sample. Ten percent of deionized water in DMEM corresponded to the highest concentrations of the agents added to the medium. The cells were cultivated in an incubator (Thermo Scientific, Forma Direct Heat CO_2_ Incubator, model 361; Thermo Fisher Scientific, Marietta, GA, USA) at 37 °C in a humidified air atmosphere saturated with 5% CO_2_ for 1, 3, and 7 days after adding the tested agents.

For an investigation of the potential cytotoxicity of nanocellulose loaded with curcumin, DC 180, and DC 300 in three concentrations (0.05, 0.1, and 0.5 mg/mL) incorporated from the water solution, the nanocellulose samples were cut into squares (8 × 8 mm^2^) and were inserted into polystyrene 48-well cell culture plates mentioned above. The samples were seeded with the cells at a density of 20,000 cells/well into 0.7 mL DMEM supplemented with 10% FBS and 40 µg/mL of gentamicin. The cells were cultivated in an incubator at 37 °C in a humidified air atmosphere saturated with 5% CO_2_ for 1, 3, and 7 days after cell seeding.

### 2.6. Cell Spreading and Cell Morphology

The morphology of the cells cultivated in the presence of curcumin, DC 180, and DC 300 in the form of a powder, or of the cells cultivated on nanocellulose samples loaded with these agents, was studied by staining the filamentous actin (F-actin) cytoskeleton and the cell nuclei. 

Before staining, the cells were fixed with 4% paraformaldehyde and were then treated with a solution of 0.2% Triton X-100 and 1% bovine serum albumin in phosphate-buffered saline (PBS) for 30 min at room temperature (RT), in order to permeabilize the cell membrane and to block the non-specific binding sites for the dyes. Then the samples were treated with a solution of 1% Tween 20 in PBS for 20 min in order to further enhance the blocking and the cell membrane permeabilization. Subsequently, the F-actin was stained with phalloidin conjugated with tetramethylrhodamine isothiocynate (TRITC) fluorescent dye (5 µg/mL, diluted in PBS), and the cell nuclei were counterstained with 4’,6-diamidin-2-fenylindol (DAPI; 1 µg/mL diluted in PBS) for 1 h at RT in the dark. All chemicals used for cell treatment were purchased from Sigma-Aldrich Co., St. Louis, MO, USA. The images of the cells were taken using an IX 51 epifluorescence microscope (objective 10×), equipped with a DP 70 digital camera (Olympus Corporation, Tokyo, Japan).

### 2.7. Cell Proliferation and Viability

The proliferation and the viability of the cells were determined by the activity of cell mitochondrial enzymes, using the Cell Titer 96^®^ AQueous One Solution cell proliferation assay (MTS, Promega Corporation, Madison, WI, USA). The assay was performed according to the manufacturer’s protocol. The amount of formazan dye produced by the cells after 2 h of incubation was quantified by measuring the absorbance. The absorbance was measured at a wavelength of 490 nm and at a reference wavelength of 700 nm, using a VersaMax ELISA microplate reader spectrophotometer (Molecular Devices Corporation, Sunnyvale, CA, USA). In the case of cells cultivated on the nanocellulose hydrogel, the nanocellulose samples were moved into fresh 48-well plates to avoid the influence of the cells adhered to the bottoms of the wells around or under the samples.

Four parallel samples were used for each experimental group and for each time interval. The experiment was performed three times. A sample without cells for each experimental group and time point was used as a control to set the background for the measured absorbance. The data were presented as the mean ± standard deviation (SD) from 4 measurements. Statistical significance was evaluated using parametric analysis of variance (ANOVA, SigmaStat 4.0, Systat Software Inc., San Jose, CA, USA) with the Tukey post hoc test for pairwise comparison. Values of *p* ≤ 0.05 were considered significant. 

### 2.8. Antimicrobial Activity of the Material

The antimicrobial activity of nanocellulose loaded with curcumin, DC 180, and DC 300 was investigated. The agents were loaded into the nanocellulose hydrogel from a water solution with an agent concentration of 0.5 mg/mL. The tested samples (square 10 × 10 mm) were immersed for 2 h into a bacterial suspension of Gram-negative *Escherichia coli* (*E. coli*, DBM 3138) with a bacteria number of 660 CFU/mL or into Gram-positive *Staphylococcus epidermidis* (*S. epidermidis*, DBM 2124) with a bacteria number of 1550 CFU/mL. Both bacterial species were obtained from the Collection of Microorganisms UCT Prague, Czech Republic. Then the bacterial suspensions were seeded on agar plates with nutritious media designated to each type of bacteria. Luria–Bertani agar (Sigma-Aldrich, St. Louis, MO, USA) was used for *E. coli* and plate count agar (Sigma-Aldrich, St. Louis, MO, USA) was used for *S. epidermidis*. The pure medium with bacteria of each strain was used as a control. The bacteria were cultivated in an incubator at 24 °C for *E. coli* and at 37 °C for *S. epidermidis*. After 24 h, a test of the antimicrobial activity of the materials was performed by the method of counting the bacterial colonies directly; 12–16 measurements were performed for each experimental group. The data were presented as the arithmetic mean ± SD. Statistical significance was evaluated using the nonparametric Kruskal–Wallis one way analysis of variance on ranks, Dunn’s method. Values of *p* ≤ 0.05 were considered significant. 

## 3. Results

### 3.1. Morphology of Bacterial Nanocellulose

**Figure 1:**
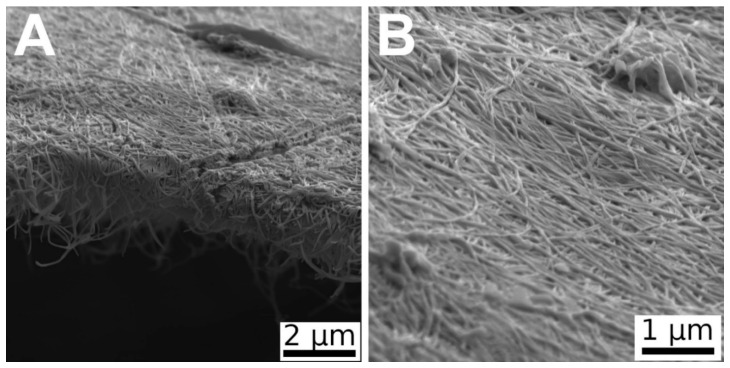
SEM images of bacterial nanocellulose cultivated for 7 days and then washed and dried with 96% ethanol. Side view (**A**) and view from above (**B**). Magnification 20× (A) and 40× (B).

The bacterial nanocellulose consisted of randomly-oriented nanofibers. The diameter of the fibers varied from 90 to 160 nm (Figure 1). 

### 3.2. Thermal Decomposition of Curcumin 

**Figure 2:**
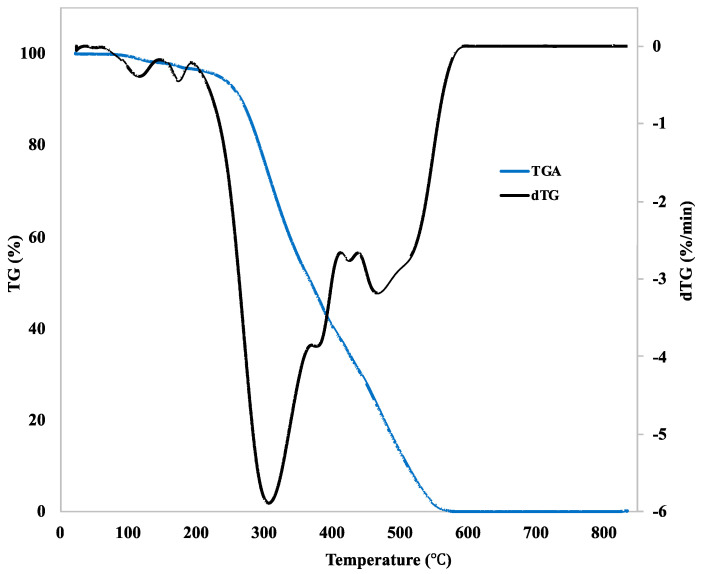
TGA and dTG curves of pure curcumin.

The thermal decomposition, manifested by a change in the curcumin mass, was studied by thermogravimetric analysis (TGA) (Figure 2). The first weight loss stage was observed at about 118 °C. This endothermal action was probably caused by crystal bound water evaporation. At about 174 °C (melting point of curcumin), no significant weight loss was detected. Clearly, the curcumin started to decompose above 200 °C. Maximum curcumin degradation was detected at a temperature of about 310 °C. It is clear from Figure 2 that the curcumin decomposition continued with a further increase in temperature, and at a temperature of 585 °C no curcumin mass remained.

**Figure 3:**
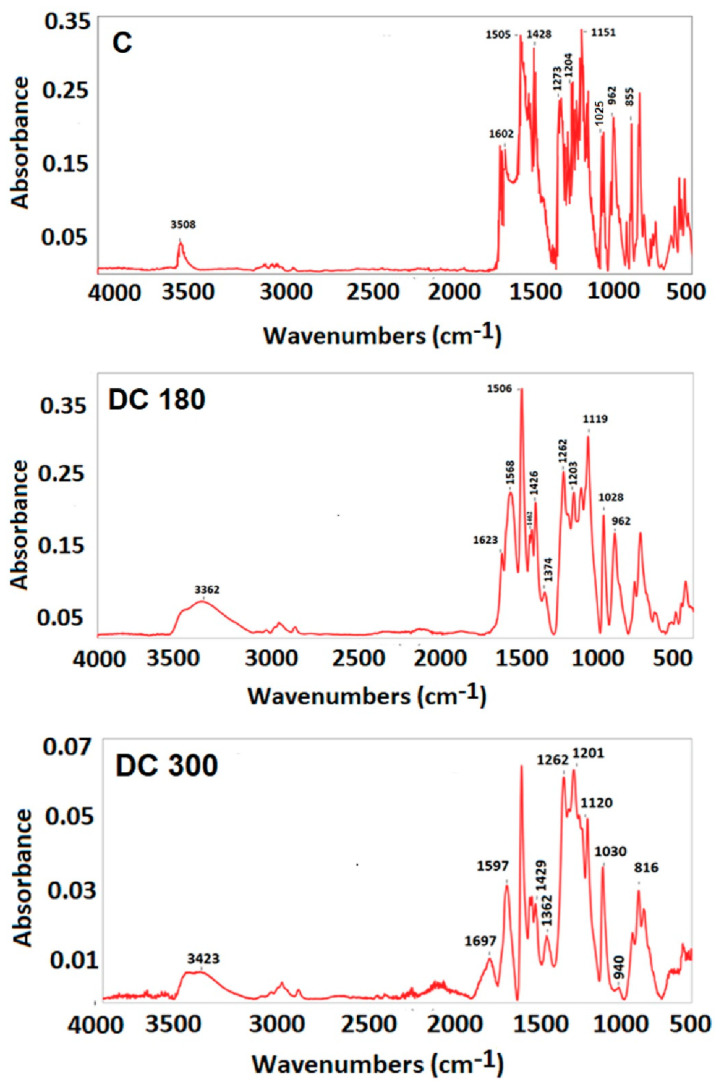
The FT-IR spectrum of untreated curcumin powder (C) and of curcumin degraded at 180 °C (DC 180) or at 300 °C (DC 300).

The characteristic functional groups occurring during thermal decomposition were studied by FT-IR. The upper part of Figure 3 represents the FT-IR spectrum of the unmodified curcumin (C). The peak at 3508 cm^−1^ was assigned to the stretching vibration of the O–H group present in the curcumin molecule. The peak at 1602 cm^−1^ indicated the C=C symmetric stretching vibration in the aromatic ring. The stronger peak at 1505 cm^−1^ could probably be assigned to the C=O bond with double bond conjugation. The peak at 1428 cm^−1^ indicated CH_2_ bending bond (deformation vibration). The enol peak was attained at 1273 cm^−1^, while the almost equally strong peak at 1204 cm^−1^ indicated the presence of phenol in the curcumin molecule. The sharp peak at 1153 cm^−1^ represented the C–CH in-plane bending vibration in phenol. Stretching C–O vibration in alkyl aryl ether was detected at 1025 cm^−1^. The sharp peak at 962 cm^−1^ was assigned to C=O and C–OH in-plane bending on the enol carbon. The two equally strong peaks at 855 cm^−1^ and at 814 cm^−1^ were attributed to hydrogen vibration, located at the 1, 2, 4-position in the aromatic ring. In the case of DC 180, the FT-IR spectrum, shown in the middle part of Figure 3, contained many spectral features similar to the features for unmodified curcumin. It was found that the intensity of the peaks at position 1203 cm^−1^ and at position 1119 cm^−1^ changed. In comparison with the unmodified curcumin, it was found that DC 180 had an inverted intensity of those peaks. This may indicate degradation of the curcumin and a change in the representation of functional groups. It was also noted that the peak at 1374 cm^−1^, which was assigned to in-plane enol C–OH vibration, started to separate and to be more noticeable in the spectrum. The intense sharp peak at 962 cm^−1^ could be attributed to the presence of ferulic acid, which characterizes C=O and C–OH bonds. However, no characteristic peaks for the aldehyde group were detected. The most characteristic peak for aldehyde, which is typically located at around 2700 cm^−1^, may have had lower intensity than the stretching C–H vibrations in the range of 2971–2849 cm^−1^, which could mask the aldehyde peak. The lower part of Figure 3 represents the FT-IR spectrum of DC 300. A new peak at 1697 cm^−1^ was detected, which was assigned to carbonyl vibration, and may be attributed to one of the degradation products. The peak at 1374 cm^−1^ separated more than in the case of DC 180. The characteristic peak for ferulic acid moved to 940 cm^−1^. These changes in peak position and in peak intensity confirmed a higher degree of degradation with increased temperature. A summarization of all important FT-IR data is given in Table S1 in the Supplementary Information.

**Figure 4:**
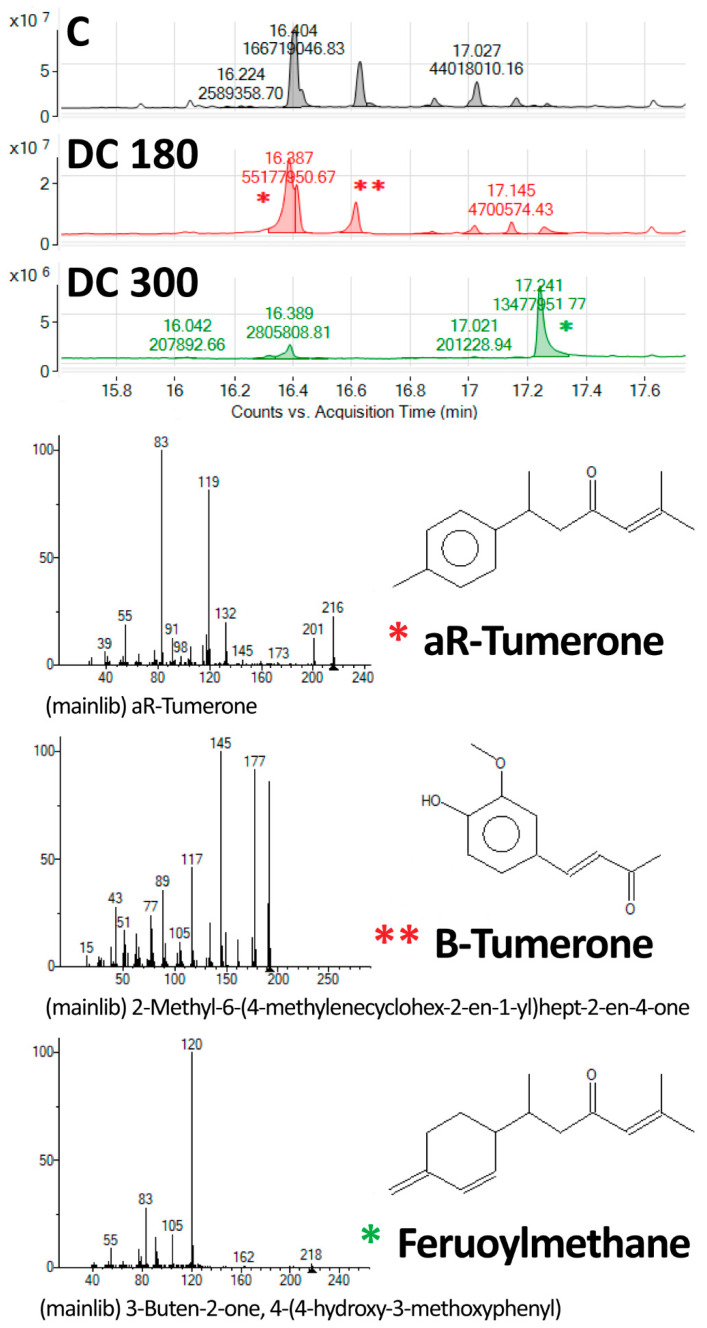
GS–MS analysis of untreated curcumin powder (C), and curcumin degraded at 180 °C (DC 180) or at 300 °C (DC 300). The main products of curcumin degradation were aR-tumerone (*), B-tumerone (**), and feruoylmethane (*). Upper part: total counts between 15.8 and 17.6 minutes of the acquisition time; lower part: details of the peaks with systematic chemical formulas and schemes of the molecules of curcumin and its degradation products.

The components formed during the thermal decomposition of curcumin were further identified by GS–MS. aR-Tumerone with its isomers, vanillin and feruloylmethane, were compounds found as products of curcumin degradation at 180 °C. However, with an increase in temperature to 300 °C, the concentration of tumerone and vanillin decreased rapidly, and feruloylmethane was detected as the main degradation product of curcumin at 300 °C (Figure 4). 

### 3.3. Cytotoxicity of the Materials

#### 3.3.1. Curcumin and its Degradation Products in the Culture Medium

**Figure 5:**
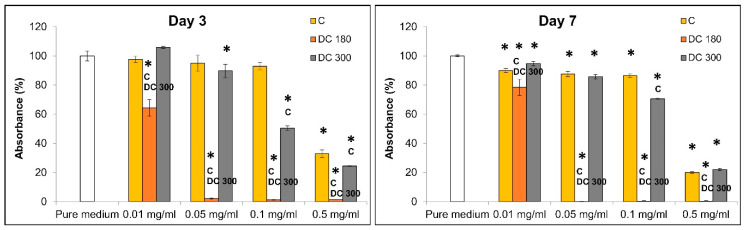
Mitochondrial activity of human dermal fibroblasts grown in a pure cultivation medium and in media with unmodified curcumin (C), or with curcumin degraded at 180 °C (DC 180) or at 300 °C (DC 300) in various concentrations (0.01, 0.05, 0.1, and 0.5 mg/mL) on days 3 and 7 after adding the agent. Arithmetic mean ± SD from 4 measurements, ANOVA, Student–Newman–Keuls method. Statistical significance (*p* ≤ 0.05; depicted above the columns): * compared with cells cultivated in the pure medium; **C** or **DC 300** compared with cells cultivated in the medium with C or DC 300 of the same concentration.

**Figure 6:**
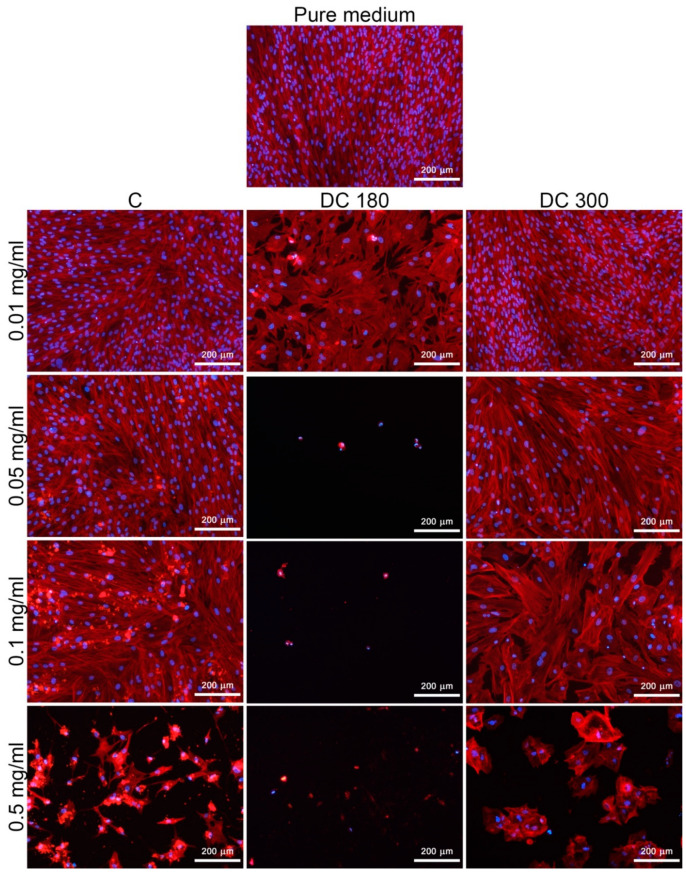
Morphology of human dermal fibroblasts cultivated in a pure cultivation medium and in media with unmodified curcumin (C), or with curcumin degraded at 180 °C (DC 180) or at 300 °C (DC 300) in various concentrations (0.01, 0.05, 0.1, and 0.5 mg/mL) on day 7 after adding the agent. The cells were stained with phalloidin-TRITC (red; F-actin cytoskeleton) and with DAPI (blue; cell nuclei). Olympus IX 51 microscope, obj. 10×, DP 70 digital camera.

The cytotoxicity of unmodified curcumin, DC 180, and DC 300 powder in various concentrations added into the culture medium was studied on human dermal fibroblasts in three time intervals by measuring the cell mitochondrial activity and by observing the cell morphology (Figure 5, Figure 6 and Figures S1 and S2). The results showed that the cytotoxicity of the agents depended on their concentrations and on the type of agent. The cytotoxicity of the agents increased with increasing concentration of the agent, mainly in the case of DC 180. The highest cytotoxicity, indicated by low cell mitochondrial activity, was observed in cells cultivated in the presence of DC 180 (Figure 5 and Figure S1). After 3 days or 7 days of cell cultivation, the cells were alive only in the presence of the lowest concentration of DC 180 (0.01 mg/mL). However, this concentration also affected the cell viability, proliferation, and morphology. The mitochondrial activity was significantly lower than for the untreated curcumin or for DC 300. In addition, the cell morphology was negatively influenced (Figure 6 and Figure S2). The cells were non-physiologically bigger than in the control pure medium, and there was a significantly smaller number of cells, which was mainly observed after 7 days of cultivation (Figure 6). DC 300 also showed a cytotoxic effect on cells; however, this effect was not so pronounced as in the case of DC 180. The cytotoxicity of DC 300 appeared at higher concentrations of 0.1 mg/mL and 0.5 mg/mL. At these concentrations, the mitochondrial activity of the cells was mostly significantly lower than for untreated curcumin (Figure 5 and Figure S1). Moreover, DC 300 at higher concentrations affected the cell morphology. DC 300 at a concentration of 0.5 mg/mL caused the formation of cell clusters, which were observed on day 7 after cell seeding (Figure 6 and Figure S2). Pure curcumin showed the lowest cell cytotoxicity. The apparent cytotoxic effect was observed only for the highest concentration of unmodified curcumin (0.5 mg/mL) (Figure 5 and Figure 6; Figures S1 and S2).

#### 3.3.2. Curcumin and Its Degradation Products in Bacterial Nanocellulose

**Figure 7:**
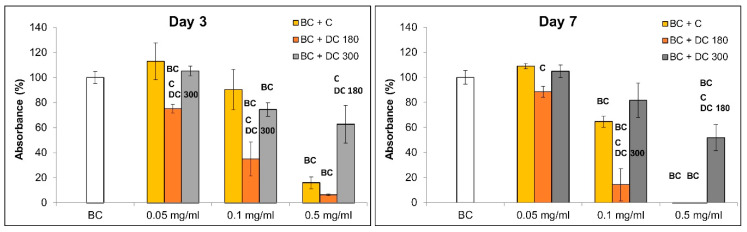
Mitochondrial activity of human dermal fibroblasts on pristine bacterial nanocellulose (BC) and on nanocellulose loaded with unmodified curcumin (BC + C), or with curcumin degraded at 180 °C (BC + DC 180) or at 300 °C (BC + DC 300 at various concentrations (0.05, 0.1 and 0.5 mg/mL) on days 3 and 7 after cell seeding. Arithmetic mean ± SD from 4 measurements, ANOVA, Student–Newman–Keuls method. Statistical significance (*p* ≤ 0.05; depicted above the columns): **BC** compared with cells cultivated on BC; **C**, **DC 180,** and **DC 300** compared with cells cultivated on BC + C, BC + DC 180, and BC + DC 300 with agents loaded at the same concentration.

**Figure 8:**
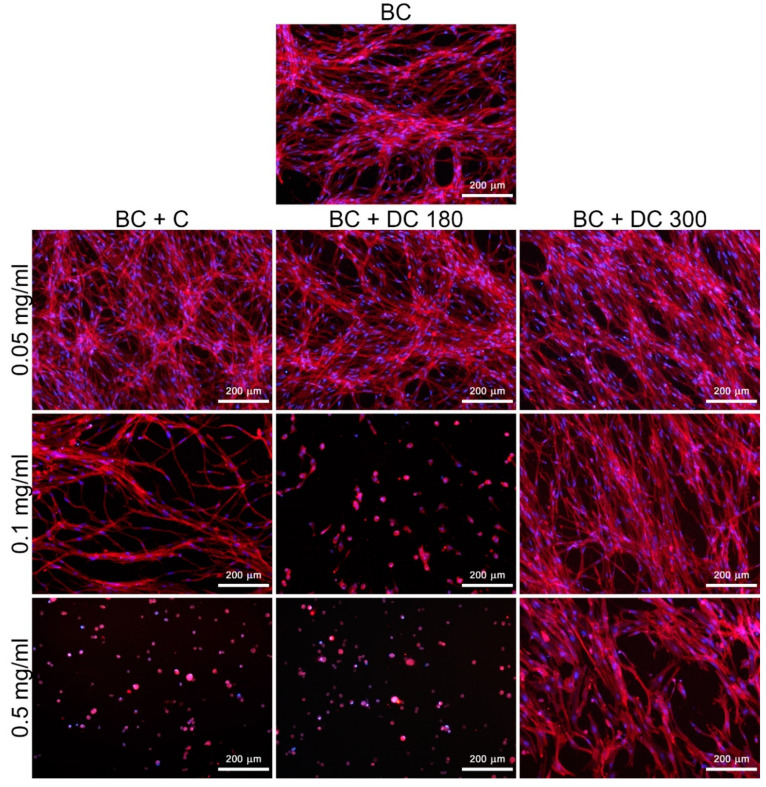
Morphology of human dermal fibroblasts on pristine bacterial nanocellulose (BC) and on nanocellulose loaded with pure curcumin (BC + C), or with curcumin degraded at 180 °C (BC + DC 180) or at 300 °C (BC + DC 300) at various concentrations (0.05, 0.1, and 0.5 mg/mL) on day 7 after cell seeding. The cells were stained with phalloidin-TRITC (red; F-actin cytoskeleton) and with DAPI (blue; cell nuclei). Olympus IX 51 microscope, obj. 10×, DP 70 digital camera.

In our study, we also investigated the behavior of the cells, particularly in terms of their adhesion, morphology, and proliferation, cultivated on nanocellulose loaded with pure curcumin, DC 180, and DC 300 powder in various concentrations for 3 and 7 days (Figure 7, Figure 8 and Figure S3). Pristine (i.e., non-loaded) bacterial nanocellulose enabled adhesion and growth of the cells. The growing cells tended to form a specific cell network on the nanocellulose surface (Figure 8 and Figure S3). Loading the nanocellulose with the agents tested here influenced the cell behavior, mainly the cell viability and cell proliferation. The influence of the agents depended on their concentration. The mitochondrial activity (Figure 7) of the cells cultivated on nanocellulose loaded with unmodified curcumin and DC 300 in the lowest concentration (0.05 mg/mL) was similar to the activity on the pristine nanocellulose, while the activity of the cells on nanocellulose loaded with DC 180 was significantly lower. At concentrations of 0.1 and 0.5 mg/mL, the nanocellulose with DC 180 showed a strong cytotoxic effect on the cells, mainly after 7 days of cell cultivation. Most of the cells seemed to be dead, as indicated by very low or zero cell mitochondrial activity and a rounded cell shape (Figure 7, Figure 8 and Figure S3). However, the cells cultivated on nanocellulose with DC 300 were able to grow, even at higher concentrations of this agent. At a concentration of 0.5 mg/mL, the cells cultivated on nanocellulose with unmodified curcumin and DC 180 were dead after 7 days of cultivation; however, the cells cultivated on nanocellulose with DC 300 were still alive. Nevertheless, their mitochondrial activity was significantly lower than on the pristine nanocellulose control. If the cells were cultivated in the presence of DC 300 powder in the culture medium (as described above), the cytotoxic effect was noticeably higher than for nanocellulose loaded with this agent. 

### 3.4. Antimicrobial Testing of the Materials

**Figure 9:**
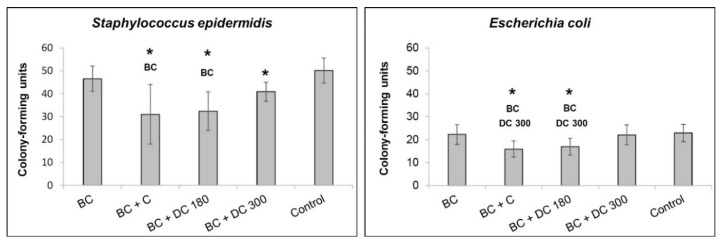
Antimicrobial activity of pristine bacterial nanocellulose (BC) and nanocellulose loaded with curcumin (BC + C), or with curcumin degraded at 180 °C (BC + DC 180) or at 300 °C (BC + DC 300) at a concentration of 0.5 mg/mL. The pure medium with bacteria was used as the control sample. The antimicrobial activity is presented for *S. epidermidis* and for *E. coli*. Arithmetic mean ± SD from 12–16 measurements for each experimental group. Nonparametric Kruskal–Wallis one way analysis of variance on ranks, Dunn’s method, statistical significance (*p* ≤ 0.05; depicted above the columns): * BC and **DC 300** compared with bacteria cultivated in the control medium, in the presence of BC and BC + DC 300, respectively.

Although pristine bacterial nanocellulose did not show any antimicrobial effect on either of the bacteria strains, i.e., *S. epidermidis* and *E. coli*, nanocellulose loaded with unmodified curcumin and with DC 180 supported fewer bacterial colonies than the control sample, i.e., bacteria in a pure medium. In the case of *S. epidermidis,* the antimicrobial effect was also observed on nanocellulose loaded with DC 300 (Figure 9). 

## 4. Discussion

According to previously published studies, curcumin is very sensitive to high temperatures, to UV light, and to pH. These varying conditions lead to curcumin decomposition [12,13,16,24]. In our study, we revealed that curcumin started to degrade at a temperature of around 180 °C. As revealed by TGA, the first relatively small weight loss in heat-treated curcumin was observed at about 118 °C. However, this loss was probably caused by crystal-bound water evaporation, because no significant further weight loss was detected at the melting point of curcumin (174 °C). At 180 °C, curcumin decomposed into aR-tumerone, vanillin, and feruloylmethane. Above this temperature, curcumin continued to degrade, and feruloylmethane was a final product of curcumin decomposition at 300 °C. Our results partly correspond with the findings of an earlier study by Esatbeyoglu et al. (2015). These authors also studied the thermal decomposition of curcumin at a temperature of 180 °C, and they found vanillin, ferulic acid, and 4-vinyl guaiacol as curcumin degradation products [13]. However, these authors roasted curcumin under different laboratory conditions and for a shorter time period (70 min) than in our study (2.5 h). This may have affected the curcumin decomposition and may have led to formation of different degradation products than we detected in our study. Chen et al. (2014) investigated the thermal decomposition kinetics and the thermodynamics of curcumin, and they reported that curcumin was clearly decomposed at a temperature of 190 °C [24]. In our study, curcumin and its degradation products were loaded into nanocellulose at a temperature of 121 °C by the autoclaving process. This higher temperature improved the incorporation of the agents into the nanocellulose hydrogel, but it had no considerable effect on the agent composition, because the loading temperature was below the curcumin decomposition temperature threshold. 

Concerning the cytotoxicity of the curcumin powder and its degradation products, the most harmful effects on the fibroblasts were caused by DC 180, while the untreated curcumin showed the lowest cytotoxicity. Moreover, the cytotoxicity depended strongly on the concentration of the curcumin or its degradation products. A study by Scharstuhl et al. (2009) revealed that curcumin at a concentration of 25 µM induced the apoptosis of fibroblasts, whereas at a concentration of 5 or 10 µM the cell viability and the cell morphology were not affected [25]. In our experiments, the lowest concentration of curcumin (0.01 mg/mL) corresponded to a dose of 25 µM. However, the curcumin in our study was probably not fully dissolved in water, so the concentration of the dissolved curcumin was lower, and the undissolved non-bioavailable part of the curcumin did not affect the cell viability. Esatbeyoglu et al. (2015) also investigated the effect of curcumin roasted at 180 °C on the viability of hepatocytes. These authors observed a cytotoxic effect with increasing concentration of the roasted curcumin, but at the same time, roasted curcumin acted as an antioxidant and as an anti-inflammatory agent [13]. According to our results, DC 180 showed high cytotoxicity on dermal fibroblasts. The main components of DC 180 were identified as tumerone, vanillin, and feruloylmethane. Previously published works have reported that aR-tumerone had a cytotoxic effect on cancer cells and could potentially be applied as an anti-cancer drug. Ji et al. (2004) examined the cytotoxic effect of aR-tumerone on various cancer cell lines, mainly on leukemia cells. They found that aR-tumerone inhibited proliferation and caused apoptosis of several leukemia cell lines [26]. Another study, by Kim et al. (2013), showed that aR-tumerone had no direct chemotherapeutic effect against leukemia cells implanted into mice, but that it had a repressive effect on lymphocytic leukemia [27].

Nanocellulose loaded with unmodified curcumin or with DC 180 exhibited a similar impact on cell viability as the powders of these agents directly added into the culture medium. This can be regarded as an indirect proof of good incorporation of both agents into the nanocellulose. Nanocellulose loaded with DC 180 showed the highest cytotoxicity, while a relatively mild impact on cell viability was observed in the cells growing on the curcumin-loaded nanocellulose, at least at lower concentrations. However, nanocellulose loaded with DC 300 showed a lower cytotoxic effect than the DC 300 powder in the culture medium. DC 300 was revealed to be the least soluble product in water, and this is what probably resulted in the poor loading of the agent into nanocellulose. The concentration of DC 300 in the nanocellulose hydrogel was probably lower than in the case of unmodified curcumin and DC 180. In addition, it is known that feruloylmethane, i.e., the main component of DC 300, is a relatively weak inhibitor of protein kinase CK2, i.e., an important anti-apoptotic enzyme in cells, and therefore it might exert only a moderate cytotoxic or antiproliferative effect on cells [28]. 

Several previous studies have declared that curcumin has antibacterial activity [5,11,15]. Our experiments have shown similar results. While pure bacterial nanocellulose did not show any antimicrobial effect on Gram-positive (*S. epidermidis)* and on Gram-negative (*E. coli*) bacteria strains, nanocellulose loaded with curcumin reduced the number of bacterial colonies in both strains. The same results were obtained for nanocellulose loaded with DC 180. However, nanocellulose loaded with curcumin and DC 180 showed a strong cytotoxic effect on dermal fibroblasts at the concentration of 0.5 mg/mL used for the antibacterial tests. Nanocellulose loaded with DC 300, which showed only a mild cytotoxic effect on dermal fibroblasts, reduced the number of colonies only in *S. epidermidis* in comparison with the bacterial culture in a pure medium.

## 5. Conclusions

In this study, we prepared bacterial nanocellulose loaded with curcumin and its degradation product for potential application in wound healing. In order to increase the bioavailability of curcumin, this compound was thermally degraded at 180 °C (DC 180) and 300 °C (DC 300). The main products of the thermal decomposition of curcumin were tumerone and vanillin at 180 °C and feruloylmethane at 300 °C. The degradation products of curcumin had a different effect on dermal fibroblasts. Although DC 180 had a strong cytotoxic effect on dermal fibroblasts, DC 300 had only a mild effect on cell viability and cell proliferation. However, both DC 180 and DC 300 were more cytotoxic than unmodified curcumin. 

The relatively high temperature (121 °C) during the autoclaving process promoted loading of the agents into the bacterial nanocellulose hydrogel. However, DC 300 was poorly soluble in water, and a smaller amount of this agent was therefore probably incorporated into the hydrogel in comparison with the unmodified curcumin and DC 180. In addition, feruloylmethane, the main component of DC 300, has been reported as a compound with relatively low cytotoxicity. As a result, the cells growing on nanocellulose loaded with DC 300 were more viable than the cells in the presence of DC 300 powder in the culture medium and were also more viable than the cells on nanocellulose incorporated with curcumin or DC 180. 

Nanocellulose loaded with curcumin or DC 180 exhibited antibacterial activity against two bacterial strains, namely *S. epidermidis* and *E. coli*. However, it also had a cytotoxic effect on dermal fibroblasts in the loading concentration (0.5 mg/mL) that was used. Nanocellulose loaded with DC 300 showed mild antibacterial activity only against *S. epidermidis*; however, the material showed almost no cytotoxicity against dermal fibroblasts. From this point of view, nanocellulose with DC 300 can therefore be considered as the most promising and most advantageous of the three combinations tested here for wound healing applications.

## Supplementary Materials

The following are available online at https://www.mdpi.com/1996-1944/13/21/4759/s1, Figure S1: Figure S1. Mitochondrial activity of human dermal fibroblasts grown in a pure cultivation medium and in media with unmodified curcumin (C), or with curcumin degraded at 180 °C (DC 180) or at 300 °C (DC 300) in various concentrations (0.01, 0.05, 0.1, and 0.5 mg/mL) on day 1 after adding the agent. Arithmetic mean ± SD from 4 measurements, ANOVA, Student–Newman–Keuls method. Statistical significance (*p* ≤ 0.05; depicted above the columns): * compared with cells cultivated in the pure medium; C or DC 300 compared with cells cultivated in the medium with C or DC 300 of the same concentration, Figure S2. Morphology of human dermal fibroblasts grown in a pure cultivation medium and in media with unmodified curcumin (C), or with curcumin degraded at 180 °C (DC 180) or at 300 °C (DC 300) in various concentrations (0.01, 0.05, 0.1, and 0.5 mg/mL) on day 1 after adding the agent. The cells were stained with phalloidin-TRITC (red; F-actin cytoskeleton) and with DAPI (blue; cell nuclei). Olympus IX 51 microscope, obj. 10×, DP 70 digital camera, Figure S3. Morphology of human dermal fibroblasts on pristine bacterial nanocellulose (BC) and on nanocellulose loaded with pure curcumin (BC + C), or with curcumin degraded at 180 °C (BC + DC 180) or at 300 °C (BC + DC 300) at various concentrations (0.05, 0.1, and 0.5 mg/mL) on day 3 after cell seeding. The cells were stained with phalloidin-TRITC (red; F-actin cytoskeleton) and with DAPI (blue; cell nuclei). Olympus IX 51 microscope, obj. 10×, DP 70 digital camera, Table S1. The functional groups responsible for IR absorption.
