# Supplementary material for: Effects of Bacterial Nanocellulose Loaded with Curcumin and Its Degradation Products on Human Dermal Fibroblasts
Source: Materials (Basel). 2020 Oct 25;13(21):4759. doi: 10.3390/ma13214759 (PMC7663456; doi:10.3390/ma13214759)
Supplement: Supplementary file 1 [file materials-13-04759-s001.pdf]

materials

Supplementary materials

Effects of Bacterial Nanocellulose Loaded with Curcumin and its Degradation Products on Human Dermal Fibroblasts

Marketa Zikmundova [1,*], Maria Vereshaka [2], Katerina Kolarova [2], Julia Pajorova [1], Vaclav Svorcik [2] and Lucie Bacakova [1]

Received: 28 September 2020; Accepted: 22 October 2020; Published: 25 October 2020

Table S1. The functional groups responsible for IR absorption.

Wave number (cm^{-1})	Primary assignment
3508	Stretching vibration of the OH
2971–2849	Stretch C–H vibrations
1697	Carbonyl vibration
1602	C=C symmetric stretching vibration in the aromatic ring
1505	C=O bond with double bond conjugation
1428	CH_2 deformation vibration
1374	In plane C–OH vibration
1153	Plane bending vibration in C_6H_5OH
1025	Stretching C–O vibration in alkyl aryl ether
962	C=O and C–OH
940	Ferulic acid
855, 814	Hydrogen vibration

Curcumin and its Degradation Products in the Culture Medium

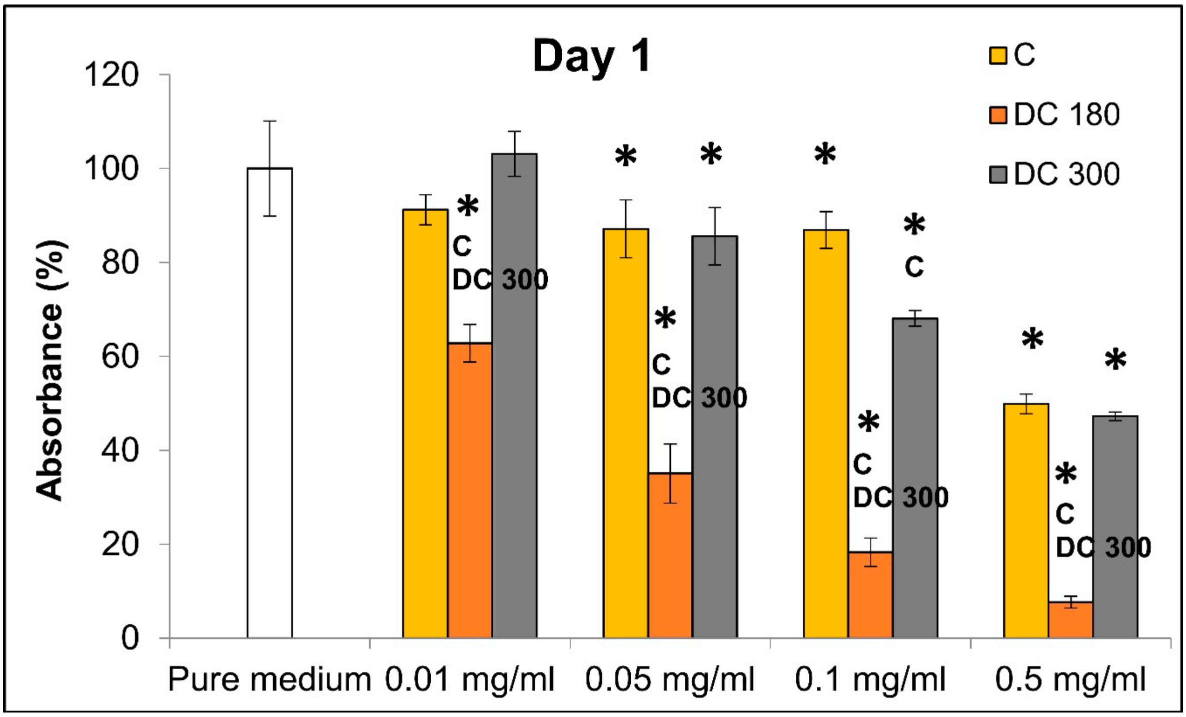

Figure S1. Mitochondrial activity of human dermal fibroblasts grown in a pure cultivation medium and in media with unmodified curcumin (C), or with curcumin degraded at 180 °C (DC 180) or at 300 °C (DC 300) in various concentrations (0.01, 0.05, 0.1, and 0.5 mg/mL) on day 1 after adding the agent. Arithmetic mean ± SD from 4 measurements, ANOVA, Student–Newman–Keuls method. Statistical significance ($p \leq 0.05$; depicted above the columns): * compared with cells cultivated in the pure medium; **C or DC 300** compared with cells cultivated in the medium with C or DC 300 of the same concentration.

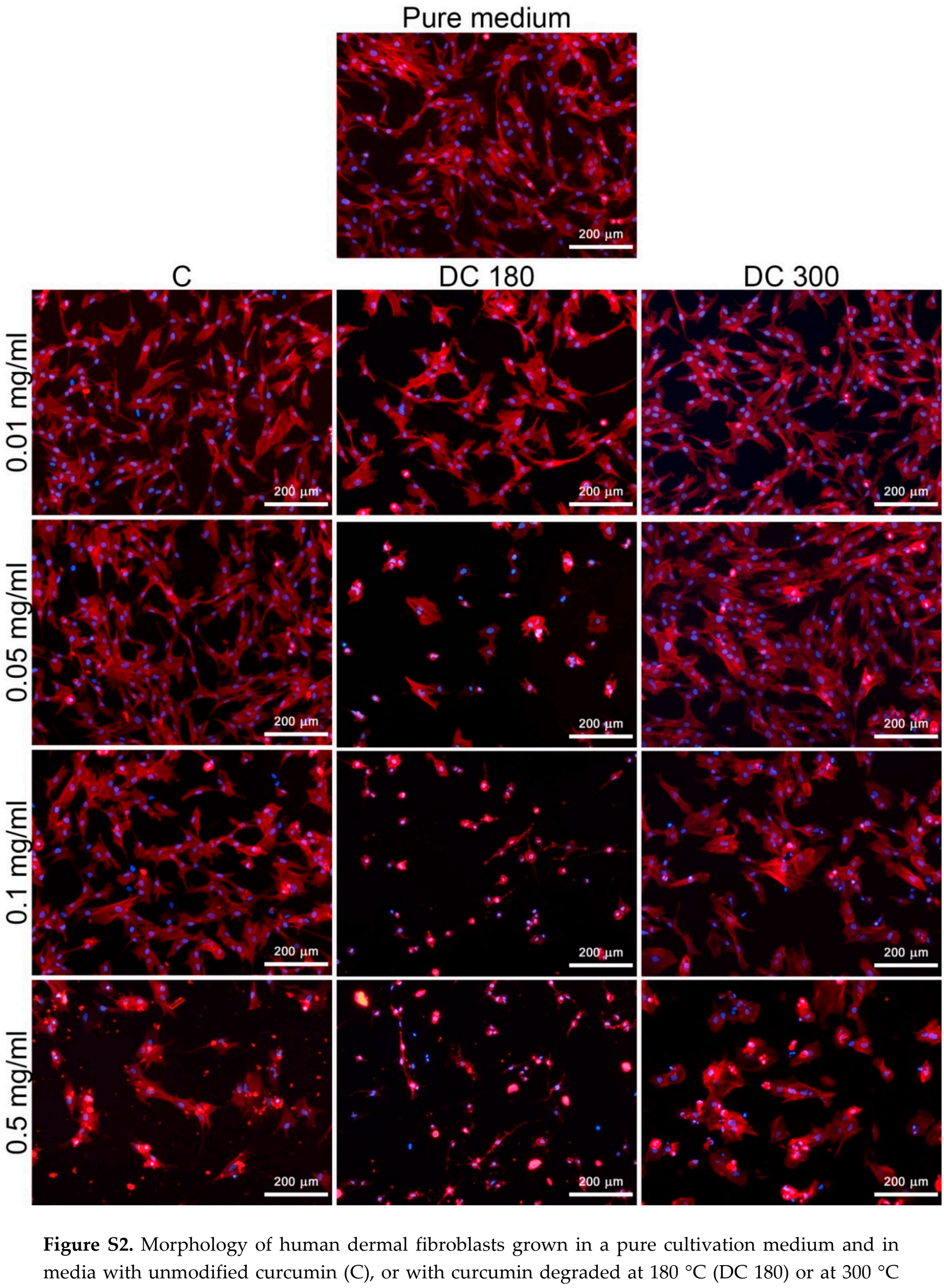

Figure S2. Morphology of human dermal fibroblasts grown in a pure cultivation medium and in media with unmodified curcumin (C), or with curcumin degraded at 180 °C (DC 180) or at 300 °C

(DC 300) in various concentrations (0.01, 0.05, 0.1, and 0.5 mg/mL) on day 1 after adding the agent. The cells were stained with phalloidin-TRITC (red; F-actin cytoskeleton) and with DAPI (blue; cell nuclei). Olympus IX 51 microscope, obj. 10×, DP 70 digital camera.

Curcumin and its Degradation Products in Bacterial Nanocellulose

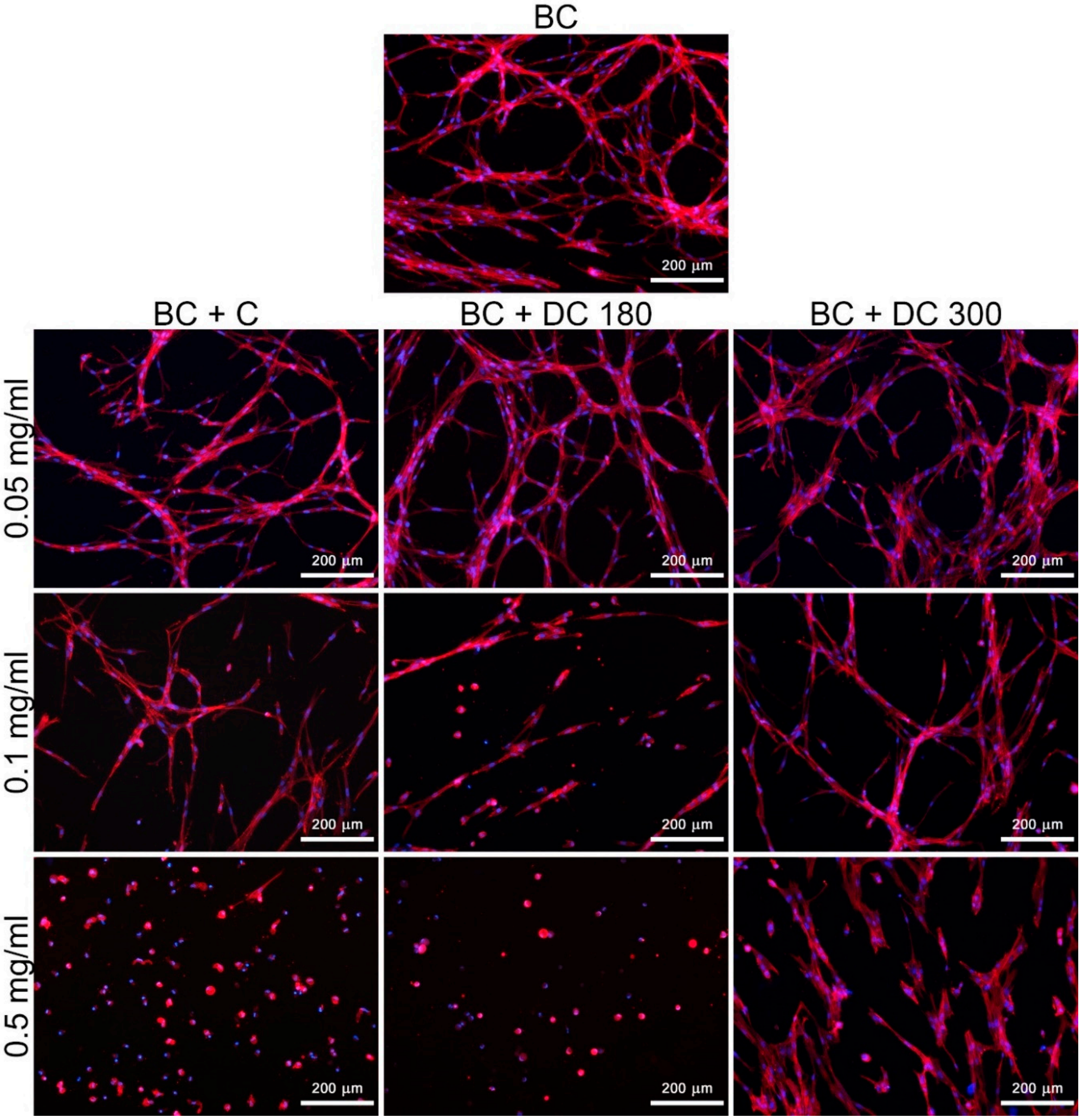

Figure S3. Morphology of human dermal fibroblasts on pristine bacterial nanocellulose (BC) and on nanocellulose loaded with pure curcumin (BC + C), or with curcumin degraded at 180 °C (BC + DC 180) or at 300 °C (BC + DC 300) at various concentrations (0.05, 0.1, and 0.5 mg/mL) on day 3 after cell seeding. The cells were stained with phalloidin-TRITC (red; F-actin cytoskeleton) and with DAPI (blue; cell nuclei). Olympus IX 51 microscope, obj. 10×, DP 70 digital camera.

© 2020 by the authors. Licensee MDPI, Basel, Switzerland. This article is an open access article distributed under the terms and conditions of the Creative Commons Attribution (CC BY) license (http://creativecommons.org/licenses/by/4.0/).
